# Self-Assembly of Aβ40, Aβ42 and Aβ43 Peptides in Aqueous Mixtures of Fluorinated Alcohols

**DOI:** 10.1371/journal.pone.0136567

**Published:** 2015-08-26

**Authors:** Sanjai Kumar Pachahara, Harikrishna Adicherla, Ramakrishnan Nagaraj

**Affiliations:** CSIR-Centre for Cellular and Molecular Biology, Uppal Road, Hyderabad, 500 007, India; University of Akron, UNITED STATES

## Abstract

Fluorinated alcohols such as hexafluoroisopropanol (HFIP) and trifluoroethanol (TFE) have the ability to promote α-helix and β-hairpin structure in proteins and peptides. HFIP has been used extensively to dissolve various amyloidogenic proteins and peptides including Aβ, in order to ensure their monomeric status. In this paper, we have investigated the self-assembly of Aβ40, Aβ42, and Aβ43 in aqueous mixtures of fluorinated alcohols from freshly dissolved stock solutions in HFIP. We have observed that formation of fibrillar and non-fibrillar structures are dependent on the solvent composition. Peptides form fibrils with ease when reconstituted in deionized water from freshly dissolved HFIP stocks. In aqueous mixtures of fluorinated alcohols, either predominant fibrillar structures or clustered aggregates were observed. Aqueous mixtures of 20% HFIP are more favourable for Aβ fibril formation as compared to 20% TFE. When Aβ40, Aβ42, and Aβ43 stocks in HFIP are diluted in 50% aqueous mixtures in phosphate buffer or deionized water followed by slow evaporation of HFIP, Aβ peptides form fibrils in phosphate buffer and deionized water. The clustered structures could be off-pathway aggregates. Aβ40, Aβ42, and Aβ43 showed significant α-helical content in freshly dissolved HFIP stocks. The α-helical conformational intermediate in Aβ40, Aβ42, and Aβ43 could favour the formation of both fibrillar and non-fibrillar aggregates depending on solvent conditions and rate of α-helical to β-sheet transition.

## Introduction

The aggregation behaviour of Aβ peptides has attracted considerable attention since its characterization in pathological conditions of Alzheimer’s disease [1–4]. The aggregation behaviour of Aβ40, Aβ42, and Aβ43 peptides *in vitro* has also been the subject of extensive investigations [5–10]. Apart from mature amyloid fibrils, oligomeric aggregates have also been identified from biological samples [11, 12]. Non-fibrillar morphology of Aβ40 and Aβ42 have been observed during *in vitro* aggregation experiments which could be either off-pathway or on-pathway aggregates [12–14]. Both off-pathway and on-pathway aggregates could be biologically relevant [15]. The presence of off-pathway aggregates could greatly influence the self-assembly of Aβ amyloid fibrils [16]. The major technical problem in setting up Aβ aggregation experiments *in vitro* is to ensure that the peptide is in the monomeric state, as Aβ peptides aggregate rapidly from aqueous solution even at low μM concentrations. In order to ensure that the Aβ peptides are monomeric in solution, various dissolution conditions have been tried. Among organic solvents, HFIP and DMSO are presumed to ensure monomeric states of the peptides even at high mM concentrations and prolonged incubation [17–20]. Fluorinated alcohols, particularly HFIP and TFE induce α-helical and/or β-hairpin structures in proteins and peptides [21–23]. Fluorinated alcohols form solvent clusters in their aqueous mixtures under a certain range of vol/vol compositions. Solvent clusters could reduce polarity around proteins/peptide molecules and thereby induce structural changes [24, 25]. The structural changes in proteins and peptides such as α-synuclein, Aβ40, Aβ42, vitronectin and K3 peptide from β_2_-microglobulin in aqueous mixtures of fluorinated alcohols have been shown to induce amyloid fibril formation [25–29]. Fluorinated alcohols, especially HFIP, has been shown to increase the rate of fibril formation in Aβ and amylin at very low concentration of solvent in aqueous media where solvent cluster formation is not evident [30, 31]. Mixtures of fluorinated alcohols and water can give useful insights in understanding aggregation pathways and intermediate structures of amyloid assembly in amyloidogenic proteins and peptides. In this study, we have examined the self-assembly of Aβ40, Aβ42, and Aβ43 from fresh HFIP stocks reconstituted under aqueous condition and aqueous mixtures of fluorinated alcohols (HFIP and TFE). We have observed the formation of fibrils and non-fibrillar aggregates in aqueous mixtures of fluorinated alcohols.

## Materials and Methods

### Materials

The peptides Aβ40, Aβ42, and Aβ43 were purchased from Peptides International (Peptide Institute, Inc., Osaka, Japan). The peptides were TFA salts. Hexafluoroisopropanol (HFIP) and trifluoroethanol (TFE) were purchased from Sigma (St. Louis, MO) and Sigma-Aldrich (St. Louis, MO), respectively. Identities of the peptides were confirmed by matrix-assisted laser desorption/ionization time-of-flight mass spectrometry.

### Peptide solutions

HFIP (200 μl) was added to the vials having lyophilized peptides. The concentrations of the peptides were estimated by recording absorption at 280 nm. Molar extinction coefficient of 1280 M^-1^ cm^-1^ at 280 nm was used to calculate the concentrations. Peptides Aβ40, Aβ42, and Aβ43 were dissolved in HFIP at 1.0, 0.8, and 0.8 mM concentrations, respectively. Peptide solutions in aqueous mixtures of HFIP and TFE were tightly capped in Axygen microcentrifuge tubes during the periods of incubation except when evaporation of organic solvent was required.

### Transmission electron microscopy (TEM)

Peptide solutions were placed on a carbon-coated Formvar 200-mesh copper grid. After 2 minutes, solvent was blotted out by touching the Whatman filter paper at peripheral part of grids. Then, grids were stained with saturated uranyl acetate solution which was blotted out after 30 seconds. Images were captured using JAM-2100 LaB6 transmission electron microscope (JEOL, Tokyo, Japan) at 100 kV. Dimensions of structures were measured with the help of software digital micrograph (Gatan, Inc.).

### Thioflavin T fluorescence spectroscopy

Thioflavin T (ThT) fluorescence spectra were recorded on Fluorolog-3 Model FL3-22 spectrofluorimeter (Horiba Jobin Yvon, Park Avenue Edison, NJ, USA). Peptide solutions in HFIP were either diluted or dried peptide films were reconstituted in aqueous conditions for aggregation reactions. ThT spectra of samples were recorded in 10 μM ThT solution in 10 mM phosphate buffer, pH 7.4 (PB). Briefly, peptides were diluted or reconstituted to 20, 10, and 5 μM peptide concentrations for Aβ40, Aβ42, and Aβ43, respectively in 10 μM ThT solution in PB. Blank ThT spectra were recorded by adding same volumes of cosolvents in 10 μM ThT solutions in buffers. The excitation wavelength was set at 450 nm, slit width at 2 nm, and emission slit width at 5 nm. Intensities of ThT fluorescence in arbitrary units (AU) at 482 nm were taken from three consecutive ThT fluorescence spectra and averaged. Data presented are averages ± standard deviation (SD).

### Circular dichroism spectroscopy

Far-UV CD spectra of the peptides were recorded on a Jasco J-815 spectropolarimeter (Jasco, Tokyo, Japan) or Chirascan spectropolarimeter (Applied photophysics, Surrey, UK). Spectra were recorded at a concentration of 20 μM from freshly prepared HFIP stocks and peptides dissolved in deionized water. CD spectra from HFIP were recorded on Jasco J-815 whereas all other spectra were recorded on Chirascan. Fresh HFIP solutions of Aβ40, Aβ42, and Aβ43 were diluted either in PB or deionized water and CD spectra were recorded immediately after dilutions at 20, 10 and 5 μM concentrations, respectively. All the spectra were recorded in 0.1 cm path length cell using a step size of 0.2 nm, band width of 1 nm, and scan rate of 100 nm min^−1^. The spectra were recorded by averaging eight scans (Jasco J-815) or two scans (Chirascan) and corrected by subtracting the solvent/buffer spectra. Mean residue ellipticity (MRE) was calculated using the formula: [θ]MRE = (Mr × θmdeg)/(100 × l × c), where Mr is mean residue weight, θmdeg is ellipticity in millidegrees, l is path length in decimeter, and c is the peptide concentration in mg ml^−1^.

## Results

### Aggregation behaviour in deionized water

**Fig 1:**
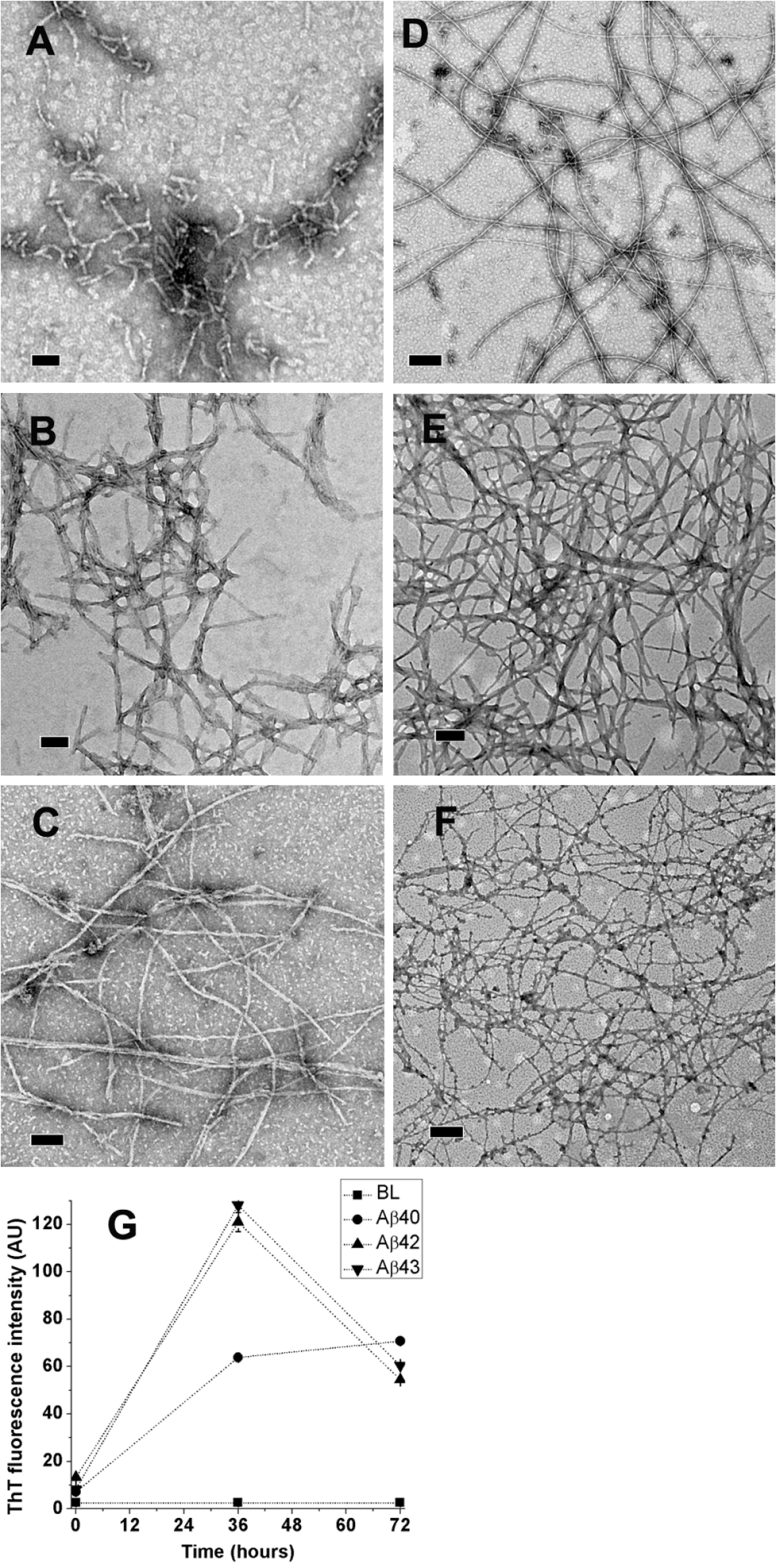
TEM images and ThT fluorescence of Aβ40, Aβ42, and Aβ43 from deionized water. Images after 36 hours (Panels A, B, and C, respectively) and 72 hours (Panels D, E, and F, respectively) of incubation at 37°C. Scale bars correspond to 200 nm. ThT fluorescence intensities at 0 hour (immediately after dissolution), 36 hours and 72 hours of incubation at 37°C are shown in panel G. Values represent average values of ThT fluorescence intensity ± standard deviation. BL denotes basal fluorescence of ThT in absence of peptides.

Dried films of Aβ40, Aβ42 and Aβ43 from freshly dissolved HFIP stocks were dissolved in deioinized water at peptide concentrations of 100 μM and incubated at 37°C in order to confirm that the peptides form amyloid fibrils as reported. After 36 hours of incubation, Aβ40 self-associates into 100–600 nm long protofibril-like structures whereas fibrillar structures of sub-micrometer to several micrometers in lengths were formed by Aβ42 and Aβ43 (Fig 1, Panels A, B, and C, respectively). After 72 hours of incubation, several micrometers long fibrillar structures were evident from all the peptides (Fig 1, Panels D, E, and F, respectively for Aβ40, Aβ42, and Aβ43). Aβ40 forms 13.35±3.64 nm thick fibrillar structures of several micrometers length along with a few protofibrils of < 200 nm length (Panel D). Aβ42 fibrils are thicker than Aβ40 fibrils (48.77±22.26 nm) as they appear to associate with each other (Panel E). Aβ43 fibrils are thinner (10.24±4.12 nm, Panel F). Thicknesses of 50 independent fibrils were measured and presented as average thickness of fibrils along with standard deviation. Intense enhancements in ThT fluorescence confirm the amyloid nature of fibrils (Panel G). Though the fluorescence spectra were recorded at same concentrations for all the peptides and ThT (10 μM, upon dilution in PB), after 36 hours of incubation, both Aβ42 and Aβ43 show considerably greater enhancement in ThT as compared to Aβ40. However, Aβ42 and Aβ43 show considerably lower enhancement in ThT fluorescence as compared to Aβ40 after 72 hours of incubation. Lower enhancement in ThT fluorescence could arise due to formation of insoluble fibrils by Aβ42 and Aβ43 on transfer from deionized water to PB as both Aβ42 and Aβ43 have faster kinetics of fibrillation as compared to Aβ40 [9].

### Aggregation behaviour in buffer solution containing 2% of HFIP

**Fig 2:**
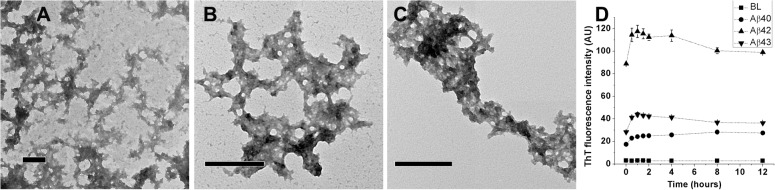
TEM images and ThT fluorescence of Aβ40, Aβ42, and Aβ43 from PB. HFIP stocks of Aβ40, Aβ42, and Aβ43 were diluted in 10 mM phosphate buffer, pH 7.4(PB) followed by 12 hours of incubation at 25°C. Aβ40, Aβ42, and Aβ43 were imaged from PB (Panels A, B, and C, respectively). Scale bars correspond to 200 nm. ThT fluorescence intensities observed over 12 hours of incubation in 2% HFIP at 25°C are shown in panel D. Values represent average values of ThT fluorescence intensity ± standard deviation. BL denotes basal fluorescence of ThT in absence of peptides.

In order to investigate the self-assembly of Aβ peptides at low concentrations of HFIP, stock solutions of Aβ40, Aβ42, and Aβ43 (1.0, 0.8, and 0.8 mM, respectively) in HFIP were diluted into PB at a concentration of 20, 10, and 5 μM, respectively. Lower concentrations of Aβ42 and Aβ43 as compared to Aβ40 were used because of their greater aggregation propensities [9, 10]. Peptides in PB were incubated at 25°C for 12 hours and imaged. TEM imaging of Aβ40, Aβ42, and Aβ43 in PB (Fig 2, Panels A, B, and C, respectively) indicate the presence of clustered aggregates. In order to evaluate whether the clusters were composed of ordered aggregates or amorphous in nature, ThT fluorescence spectra were recorded at different time points over 12 hours (Panel D). The fluorescence data indicate that ThT could bind to aggregates formed in the presence of 2% HFIP in the buffer at pH 7.4, but distinct fibrillar structures were not observed as reported previously [30]. In order to investigate the role of temperature on the formation of distinct fibrillar structures, Aβ peptides were diluted into PB at same concentrations and incubated at 37°C for 12 hours. Very similar clustered structures were observed from all the peptides in PB (data not shown). In order to identify the precursors of these clustered aggregates, peptides were imaged immediately after preparation of solutions (S1 Fig) indicating that the spherical oligomer-like structures precedes clustered aggregates.

### Aggregation behaviour in buffer containing 20% or 10% HFIP (v/v) and TFE (v/v)

**Fig 3:**
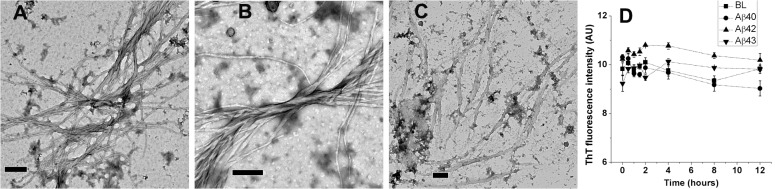
TEM images of Aβ40, Aβ42, and Aβ43 from 20% HFIP in PB. Aβ40 imaged after 2 hours of incubation at 25°C (Panel A), Aβ42 imaged after 12 hours of incubation at 25°C (Panel B), and Aβ43 imaged after 2 hours of incubation at 25°C (Panel C). Scale bars correspond to 200 nm. ThT fluorescence intensities observed over 12 hours of incubation at 25°C are shown in panel D. Values represent average values of ThT fluorescence intensity ± standard deviation. BL denotes basal fluorescence of ThT in absence of peptides.

HFIP stock solutions of Aβ40, Aβ42, and Aβ43 peptides were diluted into 20% HFIP in PB (v/v) at 20, 10, and 5 μM concentrations, respectively. Peptides in 20% HFIP were incubated at 25°C and imaged. Aβ40 and Aβ43 formed fibrils after 2 hours of incubation (Fig 3, Panels A and C, respectively) whereas Aβ42 forms fibrils after 12 hours of incubation (Fig 3, Panel B). Fibrils formed are more distinctive for Aβ40 and Aβ42 as compared to Aβ43. In order to evaluate whether the fibrils were formed in 20% HFIP solutions, ThT fluorescence spectra were recorded at different time points over 12 hours (Panel D). Intensities of ThT fluorescence are comparable to blank suggesting that the fibrils do not exist in solution. Fibrils appear to form when HFIP evaporates from the aqueous mixtures during deposition of samples for imaging.

**Fig 4:**
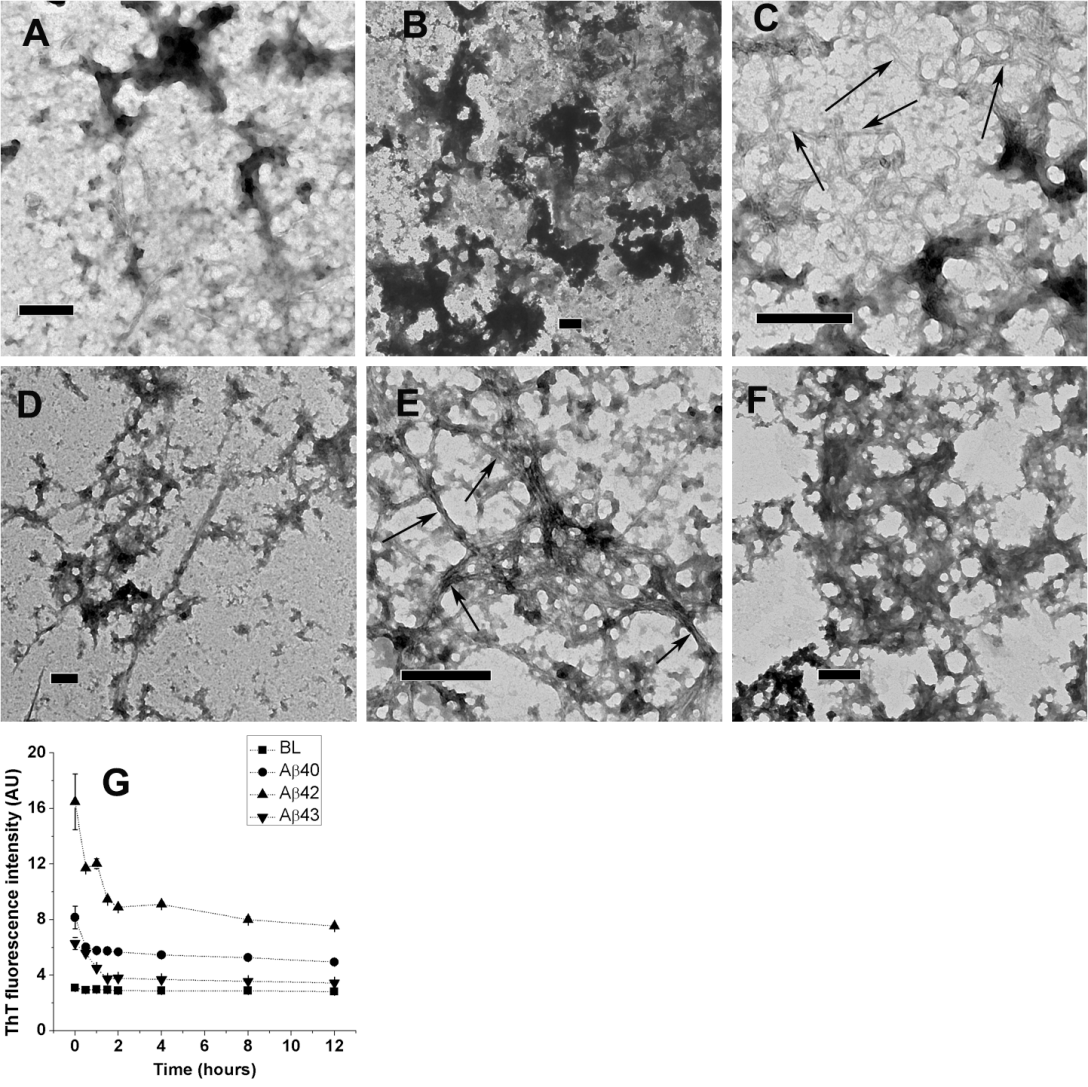
TEM images of Aβ40, Aβ42, and Aβ43 from 20% TFE in PB. Aβ40, Aβ42, and Aβ43 imaged after 12 hours of incubation at 25°C are shown in panels A, B, and C, respectively. Images from 37°C incubated stocks are shown in panels D, E, and F, respectively. Scale bars correspond to 200 nm. Fibrillar structures associated with clustered aggregates are indicated by arrows. ThT fluorescence intensities observed over 12 hours of incubation at 25°C are shown in panel G. Values represent average values of ThT fluorescence intensity ± standard deviation. BL denotes basal fluorescence of ThT in absence of peptides.

Calculated volumes of fresh HFIP stock solutions of Aβ40, Aβ42, and Aβ43 peptides were dried and dissolved in TFE followed by addition of PB to make 20% TFE in PB (v/v) at 20, 10, and 5 μM peptide concentrations, respectively. Peptides in 20% TFE were incubated at 25 and 37°C, and imaged after 12 hours of incubation. Aβ40, Aβ42, and Aβ43 formed clustered aggregates at 25°C (Fig 4, Panels A, B, and C, respectively) and 37°C (Fig 4, Panels D, E, and F, respectively). Fibrillar structures associated with clustered aggregates were observed for Aβ43 incubated at 25°C (Panel C, indicated by arrows) and Aβ42 incubated at 37°C (Panel E, indicated by arrows). ThT fluorescence spectra recorded at different time points over 12 hours at 25°C (Panel G) indicate small enhancement in ThT fluorescence suggesting the presence of a small fraction of ordered aggregates in solution. In order to identify the precursors of these clustered aggregates, peptides were imaged immediately after preparation of solutions (S2 Fig) Imaging of Aβ40 indicates isolated spherical oligomer-like structures, whereas spherical oligomers associated with each other were evident in case of Aβ42 and Aβ43 indicating that spherical oligomers precedes clustered aggregates and faster rate of formation of clustered aggregates in the case of Aβ42 and Aβ43.

**Fig 5:**
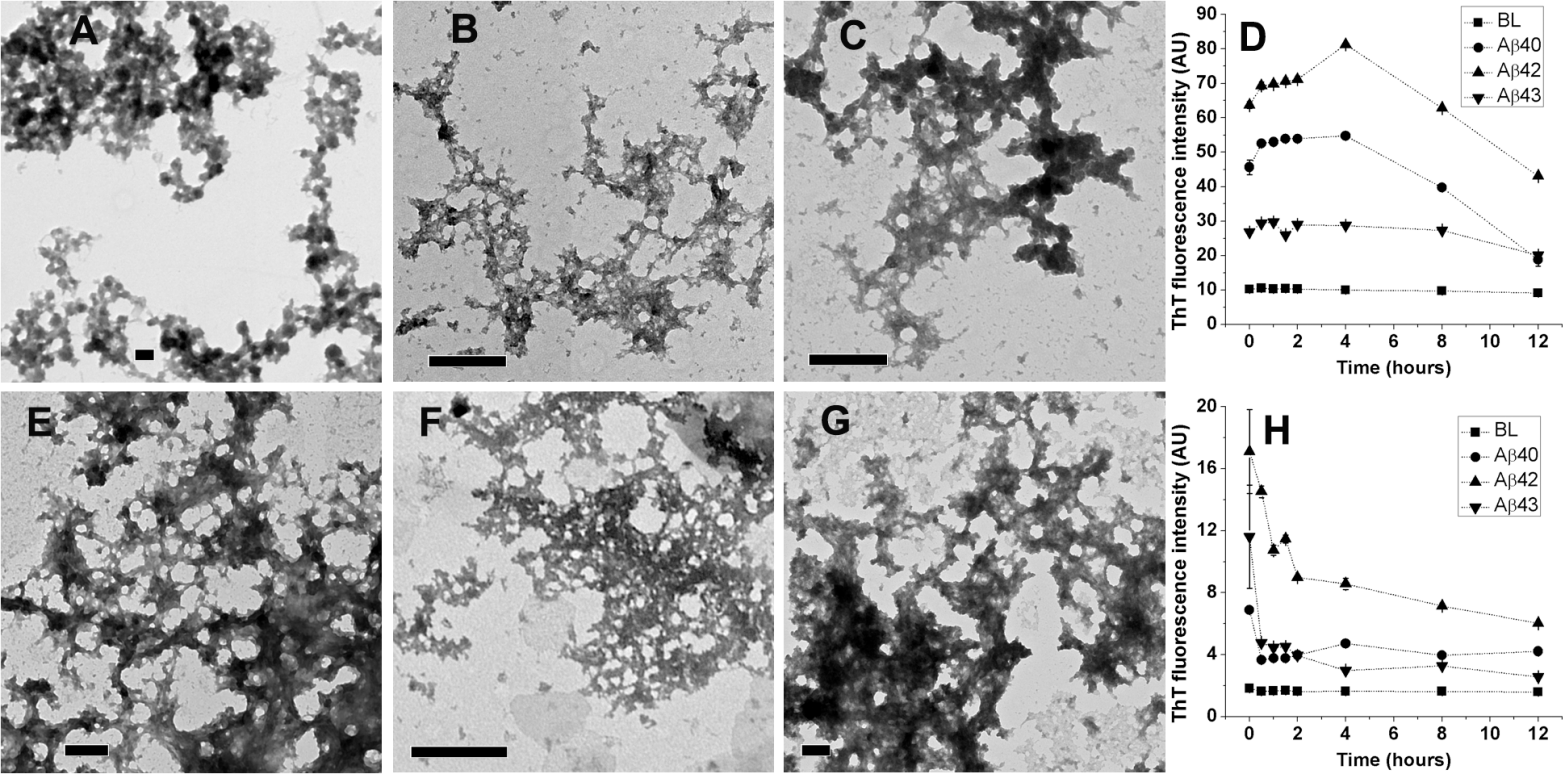
TEM images of Aβ40, Aβ42, and Aβ43 from 10% HFIP and 10% TFE in PB. Aβ40, Aβ42, and Aβ43 imaged after 12 hours of incubation at 25°C from 10% HFIP (panels A, B, and C, respectively) and 10% TFE (Panels E, F, and G, respectively). Scale bars correspond to 200 nm. ThT fluorescence intensities observed over 12 hours of incubation at 25°C in 10% HFIP and 10% TFE are shown in panels D and H, respectively. Values represent average values of ThT fluorescence intensity ± standard deviation. BL denotes basal fluorescence of ThT in absence of peptides.

HFIP stock solutions of Aβ40, Aβ42, and Aβ43 peptides were diluted into 10% HFIP in PB (v/v) at 20, 10, and 5 μM concentrations, respectively. 10% TFE stocks were prepared at the same concentration of peptides by drying and dissolution of HFIP stocks in TFE followed by addition of PB. Peptide solutions were incubated at 25°C for 12 hours prior to imaging. Aβ40, Aβ42, and Aβ43 peptides formed similar clustered aggregates from both 10% HFIP (Fig 5, Panels A, B, and C, respectively) and 10% TFE (Fig 5, Panels E, F, and G, respectively). ThT fluorescence spectra recorded over 12 hours in 10% HFIP (Panel D) and 10% TFE (Panel H) indicate greater ThT fluorescence enhancement in 10% HFIP as compared to 10% TFE. Among Aβ40, Aβ42, and Aβ43, Aβ42 showed considerably higher ThT fluorescence as compared to Aβ40 and Aβ43 under both the solvent conditions. ThT fluorescence indicate rapid aggregation of peptides immediately after solutions are prepared (0 hour) under both the solvent conditions and the reduction in ThT fluorescence (after 4 hours in case of 10% HFIP and 0.5 hours in case of 10% TFE) at later time points suggests formation of insoluble aggregates. In order to identify the precursors of these clustered aggregates, peptides were imaged immediately after preparation of solutions (S3 Fig) Peptides formed clustered aggregates from 10% HFIP when imaged immediately after preparation of solutions, whereas distinct spherical oligomers were evident from Aβ40 and Aβ43 in 10% TFE indicating faster kinetics of formation of clustered aggregates in 10% HFIP as compared to 10% TFE in case of Aβ40 and Aβ43.

**Fig 6:**
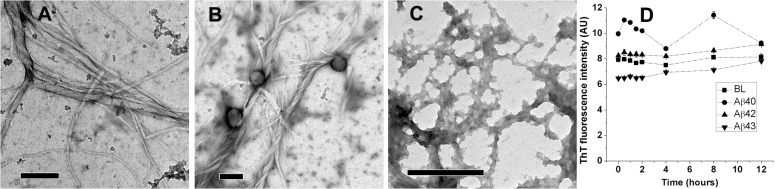
TEM images of Aβ40, Aβ42, and Aβ43 from 50% HFIP in PB. Aβ40, Aβ42, and Aβ43 imaged after 10 days of incubation at 25°C (Panels A, B, and C, respectively). Scale bars correspond to 200 nm. ThT fluorescence intensities observed over 12 hours of incubation at 25°C are shown in panel D. Values represent average values of ThT fluorescence intensity ± standard deviation. BL denotes basal fluorescence of ThT in absence of peptides.

HFIP stock solutions of Aβ40, Aβ42, and Aβ43 peptides were diluted into 50% HFIP in PB (v/v) at 20, 10, and 5 μM concentrations, respectively. Peptides were incubated at 25°C for 10 days and imaged. Aβ40 and Aβ42 formed distinct fibrillar structures whereas Aβ43 formed clustered aggregates (Fig 6, Panels A, B, and C, respectively). Fibrils from both Aβ40 and Aβ42 are of ~20 nm thickness and twist around each other to form thicker fibrils of 40–140 nm width. ThT fluorescence intensities recorded over 12 hours at 25°C are comparable to blanks (Fig 6, Panel D) indicating that fibrils do not exist in 50% HFIP solution. Fibrils appear to form when HFIP evaporates from the aqueous mixture upon deposition of samples for imaging.

Aβ peptides aggregate into clustered structures upon dilution from HFIP stock solutions into aqueous buffers at varying concentrations of HFIP and incubation temperature. Distinct fibrillar structures were also not observed when the HFIP dried peptide films were reconstituted in 10% or 20% TFE. Distinct fibrils were observed only when the peptides were diluted to 20% or 50% HFIP and incubated at 25°C but these fibrils do not appear to exist in solution and appear to form after deposition on TEM grids.

### Aggregation behaviour upon evaporation of HFIP from 50% aqueous HFIP solutions

**Fig 7:**
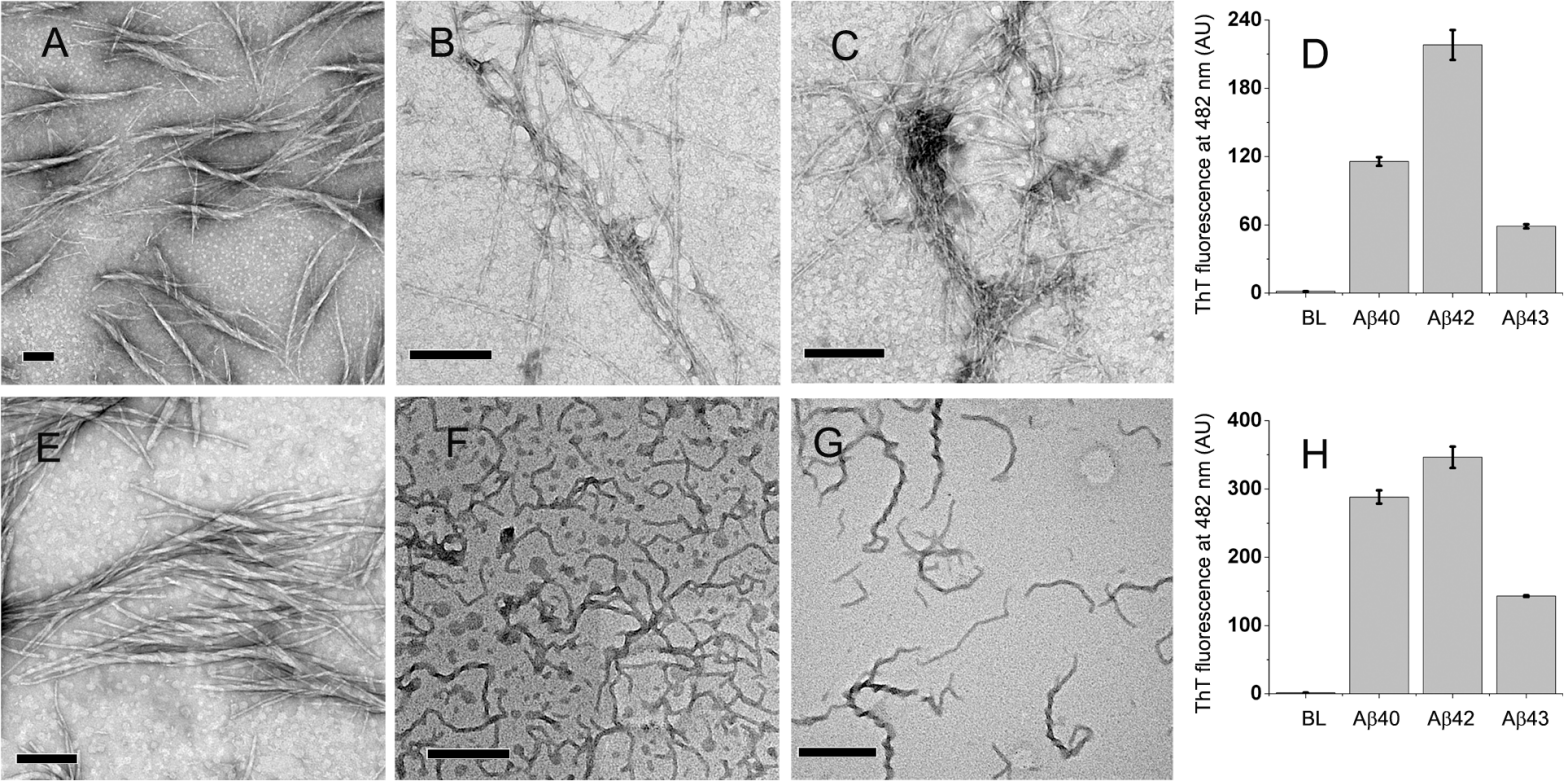
TEM images and ThT fluorescence of Aβ40, Aβ42, and Aβ43 from 50% aqueous mixtures of HFIP after complete evaporation of HFIP. Images of Aβ40, Aβ42, and Aβ43 from PB (Panels A, B, and C, respectively) and deionized water (Panels E, F, and G, respectively) after evaporation of HFIP. Scale bars correspond to 200 nm. ThT fluorescence intensities at 482 nm recorded for the peptide solutions in PB (Panel D) and deionized water (Panel H). After evaporation of HFIP from PB and deionized water, peptides were diluted two fold in 10 mM and 20 mM PB, respectively prior to recording ThT spectra. Values of ThT fluorescence at 482 nm are presented as average ± standard deviation from three consecutive spectra. BL denotes basal fluorescence of ThT in absence of peptides.

In order to confirm the formation of fibrillar structures in solution upon evaporation of HFIP, 50% aqueous HFIP stocks (in PB and deionized water; Aβ40, Aβ42, and Aβ43 stocks at 20, 10, and 5 μM concentrations, respectively) were incubated overnight at 25°C in tubes with the cap open to allow slow evaporation of HFIP. After evaporation of HFIP from 50% aqueous HFIP stocks, ThT fluorescence spectra and TEM images were recorded (Fig 7). TEM images of structures from Aβ40, Aβ42, and Aβ43 in PB after evaporation of HFIP are shown in panels A, B, and C, respectively. ThT fluorescence intensities at 482 nm for Aβ40, Aβ42, and Aβ43 in 10 mM PB at 20, 10, and 5 μM concentrations, respectively, are shown in panel D. TEM images of structures from Aβ40, Aβ42, and Aβ43 in deionized water after complete evaporation of HFIP are shown in Panels E, F, and G, respectively. ThT fluorescence intensities at 482 nm for Aβ40, Aβ42, and Aβ43 in 10 mM PB at 20, 10, and 5 μM concentrations, respectively, are shown in panel H. The observation of fibrillar structures clearly indicate that the fibrils observed in Fig 3 and Fig 6 occur due to rapid evaporation of HFIP.

### Aggregation behaviour in mixtures of deionized water and HFIP

**Fig 8:**
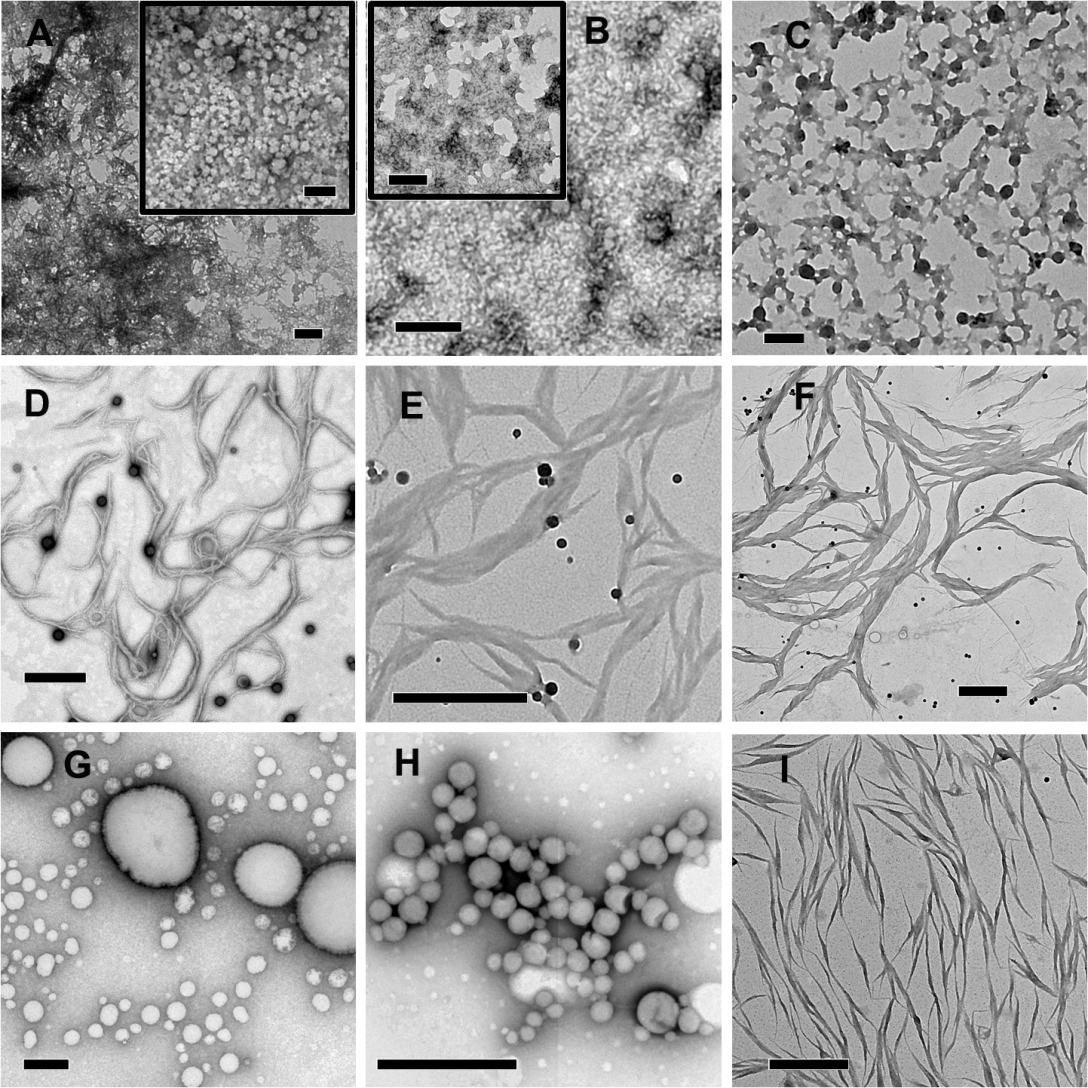
TEM images of Aβ peptides from peptide solutions in deionized water HFIP mixtures. Aβ40, Aβ42, and Aβ43 from 10% HFIP were imaged after 12 hours of incubation at 25°C (Panels A, B, and C, respectively). Aβ40, Aβ42, and Aβ43 from 20% HFIP were imaged after 2 hours of incubation at 25°C (Panels D, E, and F, respectively). Aβ40, Aβ42, and Aβ43 from 50% HFIP were imaged after 2 hours of incubation at 25°C (Panels G, H, and I, respectively). Scale bars correspond to 200 nm for panels A-C and 1 μm for panels D-I.

HFIP stock solutions of Aβ40, Aβ42, and Aβ43 peptides were diluted into mixtures of deionized water and HFIP (10%, 20%, and 50% HFIP, final concentrations of HFIP v/v) at 20, 10, and 5 μM concentrations, respectively. From 10% HFIP, Aβ40, Aβ42, and Aβ43 formed clustered aggregates (Fig 8, Panels A, B, and C, respectively) after 12 hours of incubation at 25°C. Inset in panel A indicate the presence of globular aggregates and in panel B clustered aggregates without globular structures. Fibrils were observed from all the three peptides diluted to 20% HFIP solutions incubated at 25°C for 2 hours (Fig 8, Panels D, E, and F, respectively). Lateral self-association of thinner fibrils (10–20 nm thickness) gives rise to thicker fibrils (100–300 nm thickness). From 50% HFIP after 2 hours of incubation at 25°C, Aβ40 and Aβ42 form spherical aggregates of 100 nm to 2 μm diameters whereas Aβ43 forms fibrillar structures of 50–200 nm thickness. The clustered aggregates formed from 10% HFIP solutions appears to be preceded by spherical oligomeric structures as the imaging of freshly prepared solutions indicate their presence in case of all the peptides (S4 Fig) It is likely that the structures observed from 20% and 50% HFIP may not exist in solution but formed after rapid evaporation of HFIP as observed in case of similar aqueous mixtures of HFIP in PB as observed earlier (Fig 3 and Fig 6).

### CD Spectroscopy

**Fig 9:**
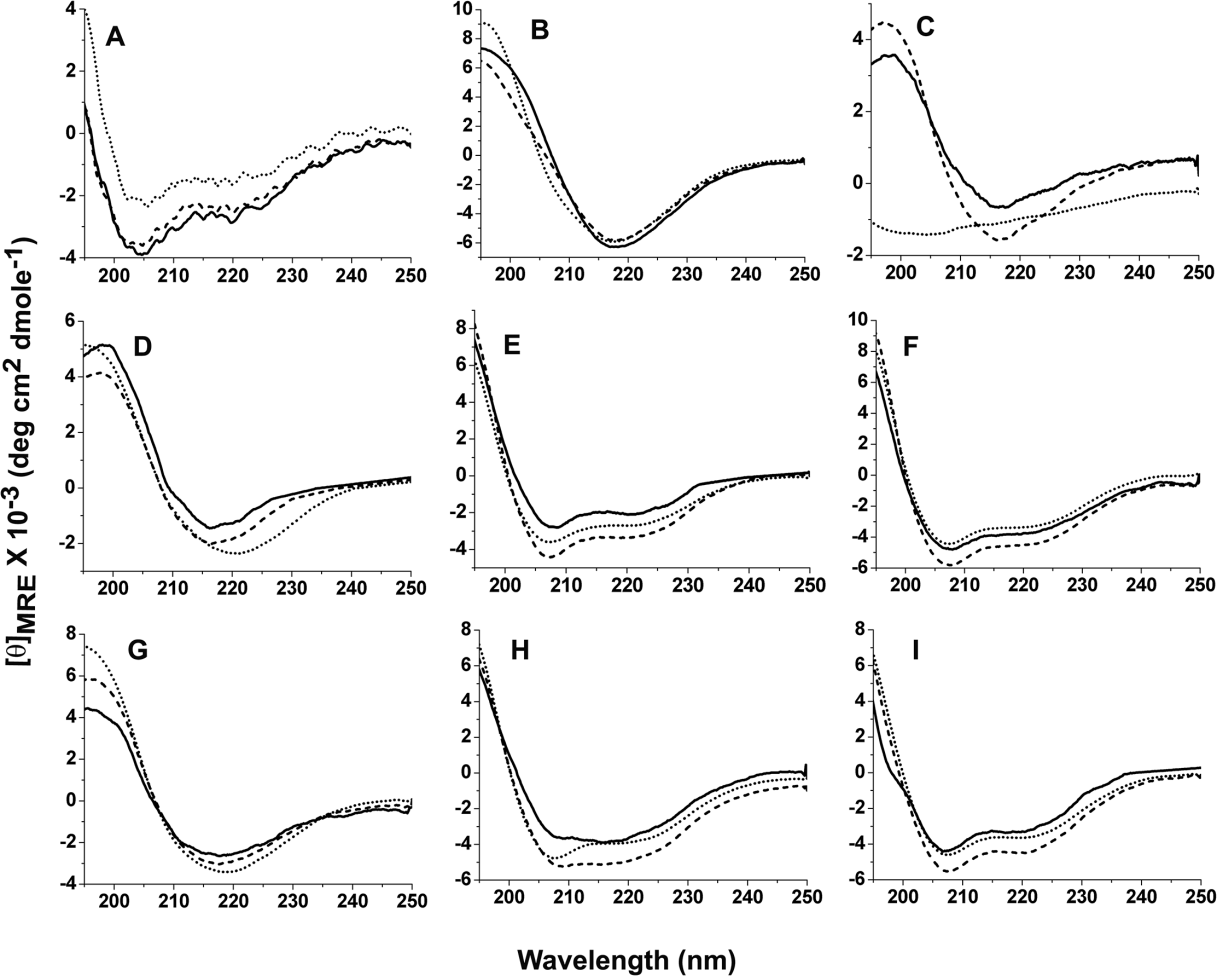
CD spectra recorded for Aβ40 (∙∙∙∙∙∙∙∙), Aβ42 (——-), and Aβ43 (—). From freshly dissolved HFIP stocks at 20 μM peptide concentrations (Panel A), from deionized water stocks incubated for 72 hours at 100 μM peptide concentrations at 37°C, after diluting to 20 μM peptide concentrations (Panel B), and from freshly prepared solutions of 2% HFIP in PB (Panel C) at 20, 10, and 5 μM concentrations for Aβ40, Aβ42, and Aβ43, respectively. From freshly prepared solutions in 10% HFIP (Panel D), 20% HFIP (Panel E) and 50% HFIP (Panel F) in PB at 20, 10 and 5 μM concentrations for Aβ40, Aβ42, and Aβ43, respectively. From freshly prepared solutions in 10% HFIP (Panel G), 20% HFIP (Panel H) and 50% HFIP (Panel I) in deionized water at 20, 10, and 5 μM concentrations for Aβ40, Aβ42, and Aβ43, respectively.

Secondary structures adopted by the peptides in HFIP, deionized water, and aqueous mixtures of HFIP were studied using far-UV CD spectroscopy. The spectra were recorded for the peptides from freshly dissolved HFIP stocks (Fig 9, Panel A), deionized water (Fig 9, Panel B), and aqueous mixture of HFIP (2% HFIP in PB, vol/vol) (Fig 9, Panel C). From freshly dissolved HFIP stocks, Peptides show a negative band at ~206 nm and a shoulder ~220–222 nm with cross-over at 196–198 nm. Qualitatively, the spectra suggest predominantly α-helical conformation. Aβ40 and Aβ42 have been shown to adopt predominantly α-helical conformation with considerable unordered conformation in fresh HFIP stocks [17]. ATR-FTIR spectra of Aβ40, Aβ42 and Aβ43 indicate predominant α-helical conformation upon drying of fresh HFIP stock solutions as reported in our previous study [32]. The presence of predominant α-helical conformation in HFIP dried films of Aβ40, Aβ42, and Aβ43 was confirmed using ATR-FTIR spectroscopy (data not shown). In deionized water, peptides adopt distinct β-sheet conformation as indicated by prominent negative band at ~218 nm and cross-over at 203–206 nm (Panel B). In 2% HFIP, Aβ42 and Aβ43 adopt distinct β-sheet conformation but Aβ40 appears to be unordered (Panel C). CD spectra were also recorded from 10%, 20% and 50% aqueous mixtures of HFIP in PB (Fig 9, Panels D, E, and F, respectively) and deionized water (Fig 9, Panels G, H, and I, respectively). Peptides adopted distinct β-sheet conformation in 10% HFIP in PB (Panel D) while distinct α-helical conformation observed in 20% and 50% HFIP in PB (Panels E and F). In 10% HFIP in deionized water, the spectra of the peptides are characteristic of β-structure whereas in 20% and 50% HFIP in deionized water, the peptides tend to adopt helical conformation (Panels G, H, and I, respectively). Presence of significant α-helical conformation in the range of 20–50% aqueous mixtures has been reported earlier for Aβ40 and Aβ42 peptides [21, 30, 33]. In order to examine the stability of α-helical conformation in aqueous mixtures of 20% and 50% HFIP in both PB and deionized water, CD spectra were recorded after 6 hours of incubation of peptide solutions at 25°C (S5 Fig). Peptides adopted α-helical conformation after incubation under all four solvent conditions suggesting that it is unlikely for peptides to self-assemble into fibrillar structures in solution under these solvent conditions.

## Discussion

Aβ40, Aβ42, and Aβ43 deposit as both fibrillar as well as non-fibrillar aggregates in the brains of Alzheimer’s disease (AD) patients [2–4, 34]. Non-fibrillar aggregates could either be neuronal cell membrane-associated form of Aβ42 which is predominantly present in diffused plaques [2] or soluble oligomers [3, 4]. As compared to the total amount of Aβ peptides in the brains of AD patients, the amount of soluble non-fibrillar Aβ directly correlates with dementia in AD patients [2, 4]. *In vitro* studies directed towards understanding Aβ self-assembly have also found a variety of aggregated species such as low and high molecular weight oligomers (LMW and HMW oligomers), amyloid-beta derived diffusible ligands (ADDLs), protofibrils, annular protofibrils, protofilaments and fibrils [35–41]. Oligomers of Aβ formed initially are unstable and undergo conformational transitions forming β-sheet rich assemblies [42]. Aβ and other amyloidogenic peptides are highly soluble in HFIP and dissolution in HFIP has been used extensively to dissociate preformed aggregates of Aβ, its short fragments, and other amyloidogenic peptides [11, 18, 43–45]. Dissolution of Aβ peptides in HFIP followed by immediate drying, has been an important step to make monomeric preparations of Aβ [17]. In some cases, the dissolved Aβ peptides are stored for prolonged periods at low temperatures [46–50]. Dissolution of Aβ40 in HFIP followed by lyophilisation has been shown to slow down the rate of fibrillization in deionized water as compared to untreated stock [51]. A recent study suggested the increased fibrillization rate of Aβ42 after HFIP treatment [52], in variance with the previous reports suggesting no significant effect of HFIP or DMSO treatment on Aβ40 and Aβ42 aggregation [13,18]. In some studies, direct reconstitution of peptide films dried from HFIP into buffer has also been employed to study Aβ fibrillization [18, 49, 53]. Structural transition from α-helix to β-sheet is a key step in Aβ aggregation and insoluble fibril formation [27, 54–56]. HFIP induces α-helical conformation in Aβ40, Aβ42, and Aβ43 peptides [17, 32].

In the present study, we have observed that Aβ40, Aβ42, and Aβ43 formed distinct amyloid fibrils in deionized water. The results are consistent with faster rate of fibrillation of longer Aβ peptides (Aβ42 and Aβ43) as reported earlier [9]. Dilution of HFIP stocks of Aβ peptides into PB containing ≤ 2% HFIP results in rapid aggregation of peptides into clustered aggregates to which ThT could bind as suggested by ThT fluorescence enhancement. However, distinct fibrillar structures were not observed. Clustered aggregates were also observed from Aβ40, Aβ42, and Aβ43 in 10% HFIP, 10% TFE and 20% TFE. Similar clustered morphology of aggregates and marked reduction in ThT fluorescence was evident upon inhibition of fibril formation or dissociation of preformed fibrils of Aβ40 and Aβ42 peptides using several organic molecules such as 3-hydroxyindole (Aβ40 and Aβ42) [57], 4-aminophenol (Aβ42) [58] and salvianolic acid B (Aβ40 and Aβ42) [59]. Incubation of Aβ40 and Aβ42 with peptide-based inhibitors of Aβ fibrillization also resulted in the formation of similar clustered aggregates that showed marked reduction in ThT fluorescence as compared to control experiments [53, 60–62].

Distinct fibrillar structures from Aβ40, Aβ42, and Aβ43 were observed from 20% HFIP when incubated at 25°C. Aβ40 and Aβ42 formed fibrils from 50% HFIP in PB whereas Aβ43 formed only clustered aggregates after incubation at 25°C for 10 days. Aβ40, Aβ42, and Aβ43 adopt α-helical conformation in both 20% and 50% aqueous mixtures of HFIP, which would not favour the formation of fibrils in solution. Moreover, ThT fluorescence enhancement over 12 hours is comparable to blank, confirming that fibrils do not exist in solution although distinct fibrillar structures are observed by EM. There is evaporation of HFIP from these solvent compositions when deposited over the grid for recording TEM images, which could lead to the reduction in percentage of HFIP in mixtures. The reduction in percentage of HFIP could induce slow conversion of α-helical to β-sheet conformation resulting in to distinct fibrillar structures whereas rapid conversion of α-helical to β-sheet conformation observed in aqueous mixtures of 2% and 10% HFIP leads to formation of clustered aggregates. Although the presence of initial α-helical conformation is important for fibril assembly [54–56], our results indicate that slow conversion of α-helical to β-sheet conformation favours fibril assembly whereas rapid conversion of α-helical to β-sheet favours formation of clustered structures. When HFIP was evaporated completely from 50% aqueous mixtures in PB and deionized water, the fibrillar structures resulting in intense ThT fluorescence were confirmed in PB and deionized water. Recently, we have reported distinct fibrillar structures formed by Aβ(16–22) and its aromatic variants in 20% aqueous mixtures of HFIP and TFE [63] Fibril formation has been reported for Aβ42 from 50% HFIP in deionized water under similar incubation time followed by complete evaporation of HFIP from the mixture but the images of fibrillar structures were not shown [64,65]. The multifunctional protein vitronectin deposits in senile plaques of AD brain and associated with systemic amyloidoses readily forms amyloid fibrils from 50% HFIP [29]. The pH of aqueous organic mixture appears to play an important role in fibril formation. Fibrils are formed by Aβ40 and Aβ42 but not by Aβ43 from 50% mixture of HFIP and PB (pH 7.4), whereas in 50% mixture of HFIP and deionized water (pH 6.4), fibrils are formed by Aβ43 but not by Aβ40 and Aβ42. These observations indicate that fibril formation by Aβ40, Aβ42 and Aβ43 peptides is sensitive to small changes in pH. From 20% HFIP, fibrils are formed at both the pH conditions but there is significant difference in the morphologies of fibrils. Aβ40, Aβ42, and Aβ43 in 10% HFIP (pH 7.4), Aβ43 in 50% HFIP (pH 7.4) and Aβ40 and Aβ42 in 50% HFIP (pH 6.4) did not form distinct fibrils suggesting that fibril formation is sensitive to the presence of solvent clusters and physicochemical properties of co-solvents. The physicochemical properties of the solvent mixtures do not stabilize amyloid competent conformers which can favour the formation of distinct Aβ fibrils. Fibrils of Aβ40, Aβ42, and Aβ43 formed from 20% and 50% HFIP have vary wide range of thicknesses indicating that lateral association and twisting of thinner fibrils (10–20 nm thickness) with each other results to formation of thicker fibrils. It suggests that thinner fibrils could have exposed hydrophobic surfaces which can stabilise lateral association and twisting of two or more fibrils giving rise to thicker fibrils as observed in case of insulin fibrils [66]. Similarly, clustered aggregates could be resulted from the self-association of oligomeric on-pathway and/or off-pathway aggregates.

Aqueous mixtures of 20% TFE are reported to promote amyloid fibril formation in Aβ, α-synuclein and β2m peptide, presumably due to the presence of dynamic organic solvent clusters [25–28]. The present study indicates formation of clustered aggregates by Aβ40, Aβ42, and Aβ43 in aqueous mixtures of 10% and 20% TFE. 10–20% HFIP has been shown to dissociate pre-existing Aβ oligomeric aggregates except trimers [11] whereas no aggregation was observed in 10% HFIP in aqueous buffer [30]. The results presented in this study indicate formation of both fibrillar and non-fibrillar aggregates depending on peptide and solvent conditions.

It is likely that low molecular weight oligomers of Aβ40, Aβ42, and Aβ43 are present in the HFIP solutions as observed earlier for Aβ40 and Aβ42 [52, 67] which could seed both on-pathway and off-pathway aggregates. A recent study has identified a 56 kDa oligomeric species of Aβ which does not dissolve in NaOH, HFIP, formic acid, urea, and guanidine [68]. Depending on the solvent conditions such as peptide concentration, temperature, time of incubation, and percentage of fluorinated alcohol in aqueous organic mixtures can either favour formation of on-pathway or off-pathway aggregates. Under some of the solvent conditions, self-assembly of on-pathway and off-pathway aggregates could be highly competitive and both types of aggregates can co-exist. α-Helical to β conformational transitions of Aβ42 in aqueous HFIP medium are reversible [69]. The self-assembly of Aβ peptides into fibrillar structures is a complex process which requires distinct conformational rearrangement of structurally dynamic monomers and oligomers [14, 70–72] resulting in the formation of fibrils and non-fibrillar clusters. Also, the aggregation steps in Aβ40, Aβ42, and Aβ43 are not identical and could follow different pathways [73] and difference in packing interactions within the fibrils could lead to polymorphic fibrils with varying stabilities [74]. The α-helical conformational intermediate in Aβ40, Aβ42, and Aβ43 could favour the formation of both fibrillar and non-fibrillar aggregates depending on the solvent conditions. Change in solvent conditions could modulate conformation or solubility of Aβ40, Aβ42, and Aβ43 which could lead to change in aggregation pathways and aggregation rates by modulating the dynamics of structural rearrangements. The use of 50% HFIP aqueous mixtures for dissolution of Aβ peptides followed by slow evaporation of HFIP could conceivably be useful in screening inhibitors of Aβ fibrillization, as the α-helical conformational intermediate in Aβ fibrillization would be accessible for longer time for inhibitors to bind with this amyloid competent conformer during slow evaporation.

## Supporting Information

S1 Fig TEM images of Aβ40, Aβ42, and Aβ43 from 2% HFIP in PB. HFIP stocks of Aβ40, Aβ42, and Aβ43 were diluted in PB and imaged immediately after preparation (Panels A, B, and C, respectively). Scale bars represent 200 nm.
(TIF)

S2 Fig TEM images of Aβ40, Aβ42, and Aβ43 from 20% TFE in PB. Freshly prepared solutions of Aβ40, Aβ42, and Aβ43 were imaged immediately after preparation (Panels A, B, and C, respectively). Scale bars represent 200 nm.
(TIF)

S3 Fig TEM images of Aβ40, Aβ42, and Aβ43 from 10% HFIP and 10% TFE in PB. Freshly prepared solutions of Aβ40, Aβ42, and Aβ43 in 10% HFIP (Panels A, B, and C, respectively) and 10% TFE (Panels D, E, and F, respectively) were imaged immediately after preparation. Scale bars represent 200 nm.
(TIF)

S4 Fig TEM images of Aβ40, Aβ42, and Aβ43 from 10% HFIP in deionized water. Freshly prepared solutions of Aβ40, Aβ42, and Aβ43 were imaged immediately after preparation (Panels A, B, and C, respectively). Scale bars represent 200 nm.
(TIF)

S5 Fig CD spectra recorded for Aβ40 (∙∙∙∙∙∙∙∙), Aβ42 (——-), and Aβ43 (—). After 6 hours of incubation of freshly prepared solutions in 20% HFIP in PB (Panel A), 50% HFIP in PB (Panel B), 20% HFIP in deionized water (Panel C), and 50% HFIP in deionized water (Panel B) at 20, 10, and 5 μM concentrations for Aβ40, Aβ42, and Aβ43, respectively.
(TIF)
