# Supplementary figures and images for: Self-Assembly of Aβ40, Aβ42 and Aβ43 Peptides in Aqueous Mixtures of Fluorinated Alcohols
Source: PLoS One. 2015 Aug 26;10(8):e0136567. doi: 10.1371/journal.pone.0136567 (PMC4550328; doi:10.1371/journal.pone.0136567)

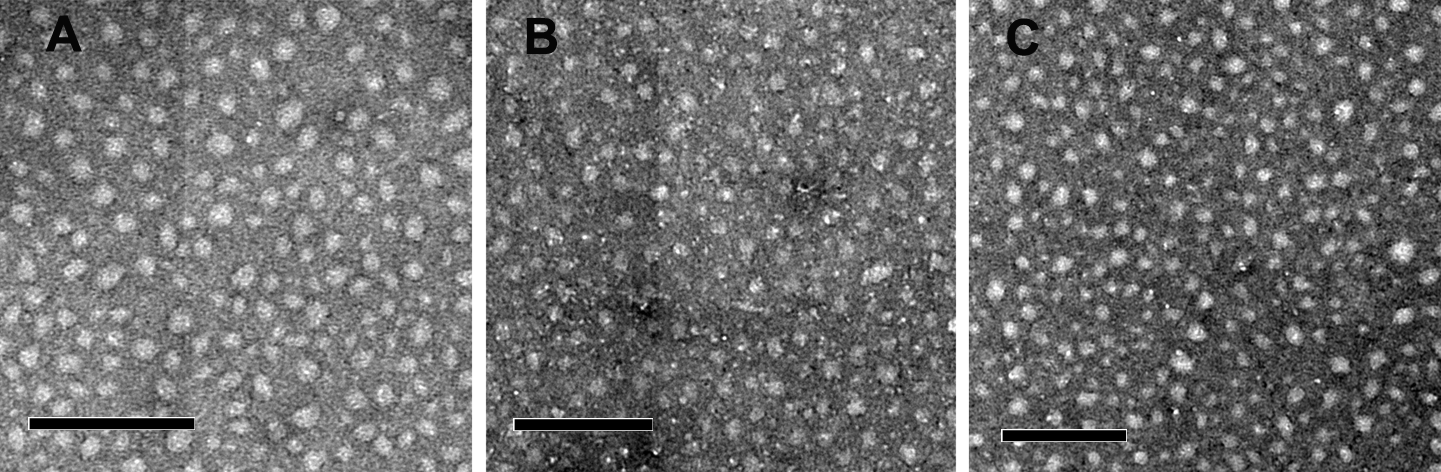

Supplement: S1 Fig — HFIP stocks of Aβ40, Aβ42, and Aβ43 were diluted in PB and imaged immediately after preparation (Panels A, B, and C, respectively). Scale bars represent 200 nm. (TIF) [file pone.0136567.s001.tif]

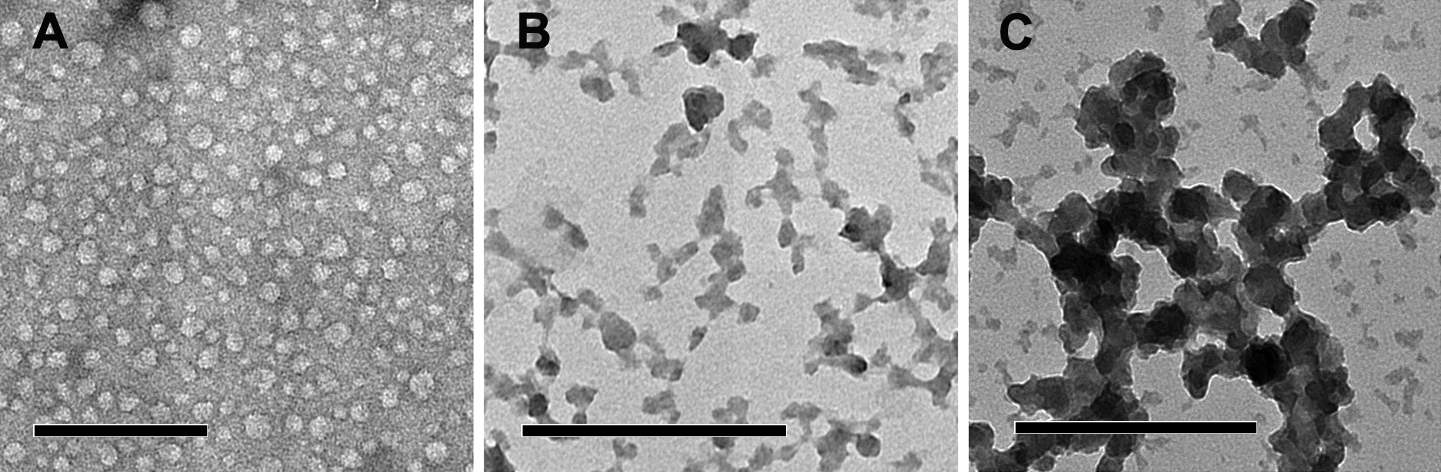

Supplement: S2 Fig — Freshly prepared solutions of Aβ40, Aβ42, and Aβ43 were imaged immediately after preparation (Panels A, B, and C, respectively). Scale bars represent 200 nm. (TIF) [file pone.0136567.s002.tif]

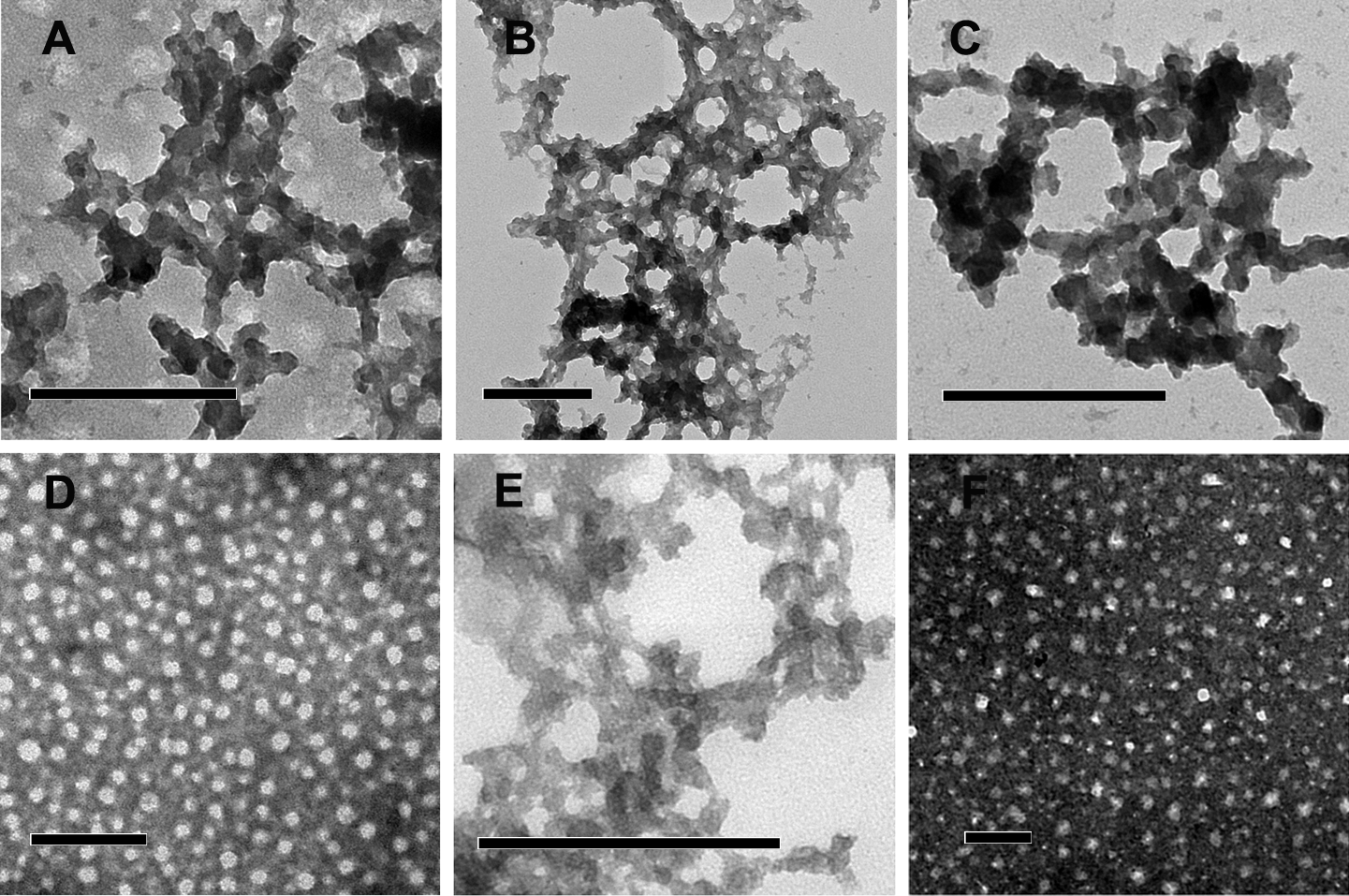

Supplement: S3 Fig — Freshly prepared solutions of Aβ40, Aβ42, and Aβ43 in 10% HFIP (Panels A, B, and C, respectively) and 10% TFE (Panels D, E, and F, respectively) were imaged immediately after preparation. Scale bars represent 200 nm. (TIF) [file pone.0136567.s003.tif]

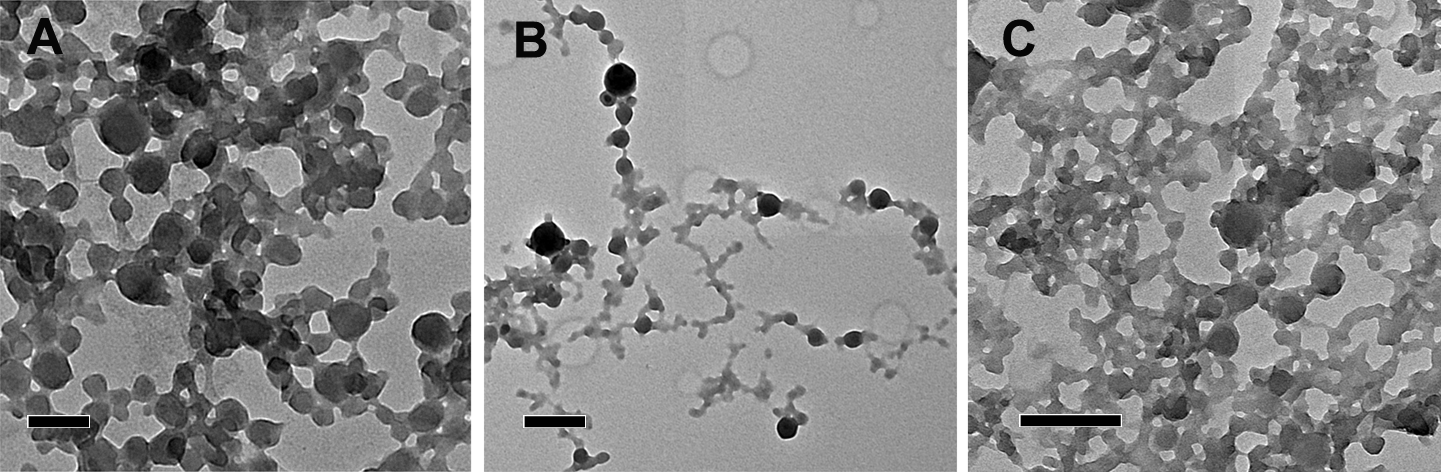

Supplement: S4 Fig — Freshly prepared solutions of Aβ40, Aβ42, and Aβ43 were imaged immediately after preparation (Panels A, B, and C, respectively). Scale bars represent 200 nm. (TIF) [file pone.0136567.s004.tif]

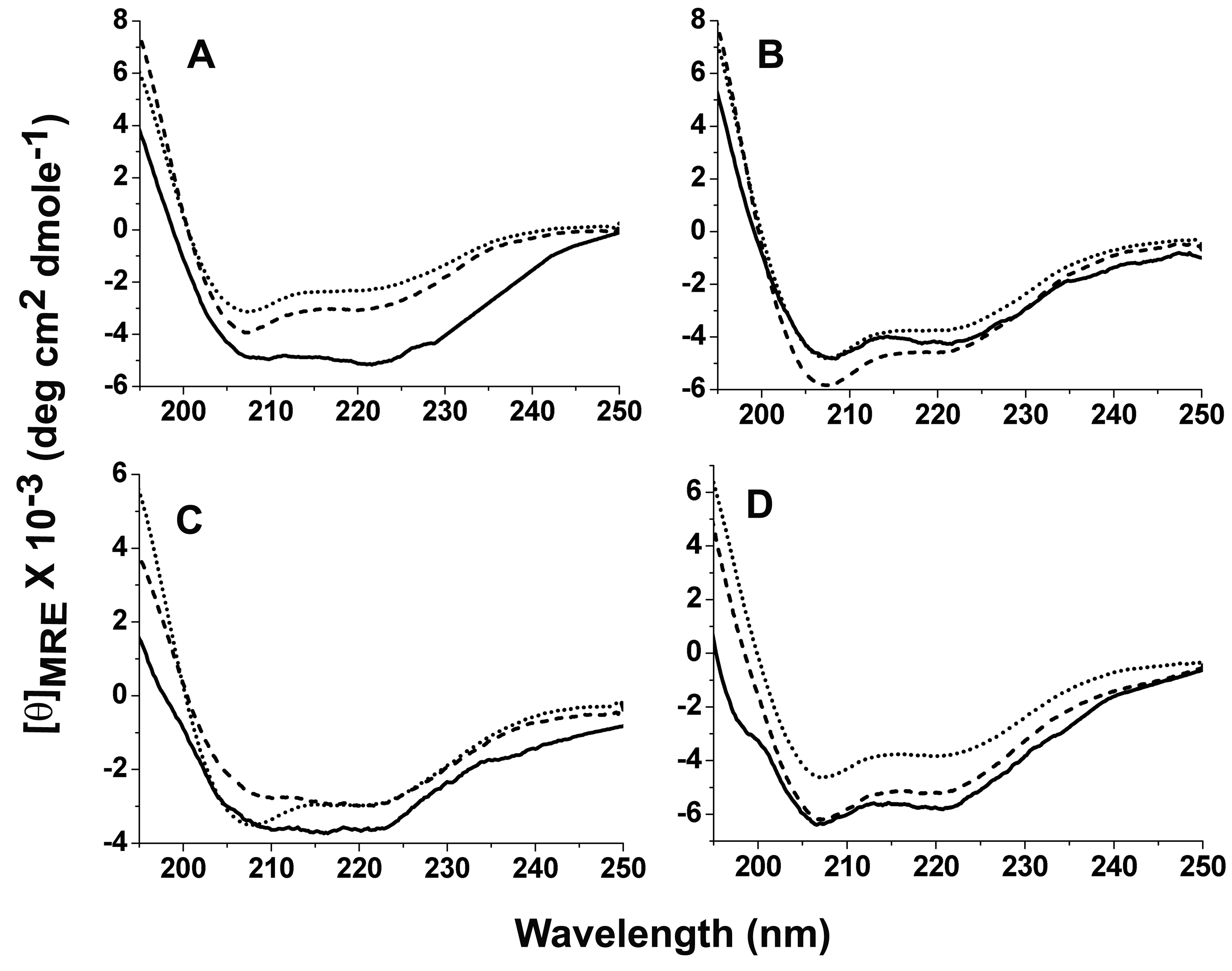

Supplement: S5 Fig — After 6 hours of incubation of freshly prepared solutions in 20% HFIP in PB (Panel A), 50% HFIP in PB (Panel B), 20% HFIP in deionized water (Panel C), and 50% HFIP in deionized water (Panel B) at 20, 10, and 5 μM concentrations for Aβ40, Aβ42, and Aβ43, respectively. (TIF) [file pone.0136567.s005.tif]
